# Modifiable ADRD risk factors among family caregivers in the United States: Results from the 2021‐2023 Behavioral Risk Factor Surveillance System

**DOI:** 10.1002/alz70860_106711

**Published:** 2025-12-23

**Authors:** Tiffany B Kindratt, Erin D. Bouldin, Monika Lopez‐Anuarbe, Aya Yoshikawa, Noah J Webster

**Affiliations:** ^1^ University of Texas at Arlington, Arlington, TX, USA; ^2^ University of Utah School of Medicine, Salt Lake City, UT, USA; ^3^ Connecticut College, New London, CT, USA; ^4^ Texas Woman's University, Denton, TX, USA; ^5^ University of Michigan, Ann Arbor, MI, USA

## Abstract

**Background:**

Alzheimer's disease and related dementias (ADRD) caregivers are a unique subgroup of adults in the US that may be at increased risk of ADRD themselves due to both unmodifiable (e.g., sex and age) and modifiable (e.g., health behaviors) risk factors. In 2024, the Lancet Commission on Dementia Prevention, Intervention and Care identified 14 potentially modifiable ADRD risk factors across the life course that contribute to a 45% reduction in population attributable risk. Our objective was to estimate and compare the prevalence of each modifiable risk factor for ADRD among ADRD caregivers, non‐ADRD caregivers, and non‐caregivers.

**Method:**

We used 2021‐2023 Behavioral Risk Factor Surveillance System data. The sample comprised 6,959 family ADRD caregivers, 57,267 family caregivers of people with other health conditions (non‐ADRD), and 256,677 non‐caregivers. Modifiable risk factors evaluated were less than college education, hearing loss, high cholesterol, hypertension, alcohol use, obesity, smoking, depression, physical inactivity, diabetes, vision loss, and social isolation. Bivariate analysis using chi square tests was conducted.

**Result:**

ADRD caregivers were significantly more likely to be 45‐64 years old (45%) and female (64%) compared to non‐ADRD caregivers (40% and 61%, respectively) and non‐caregivers (32% and 51%, respectively; *p* < .05). The prevalence of ADRD risk factors among ADRD caregivers ranged from 5% for vision loss to 60% for less than college education. ADRD caregivers were significantly more likely to have high cholesterol (45%) compared to non‐caregivers (40%) and non‐ADRD caregivers (43%). The prevalence of hypertension was significantly higher among ADRD caregivers (44%) than among non‐caregivers (40%). Conversely, ADRD caregivers had a significantly lower prevalence of less than college education (53%) and physical inactivity (21%) compared to non‐caregivers (60% and 25%, respectively) and non‐ADRD caregivers (60% and 15%, respectively; *p* < .05).

**Conclusion:**

Our findings provide the first comprehensive look at the epidemiology of modifiable ADRD risk factors among ADRD caregivers whose health is often overlooked in the medical literature. ADRD caregivers had a high prevalence of several risk factors, while other risk factors were less common compared to non‐caregivers and non‐ADRD caregivers. Interventions and efforts to reduce risk factors for ADRD (e.g., hypertension) may be particularly beneficial for ADRD caregivers.

